# ﻿The complete mitochondrial genome of *Meloeproscarabaeus* (Coleoptera, Meloidae): genome descriptions and phylogenetic inferences

**DOI:** 10.3897/zookeys.1109.81544

**Published:** 2022-07-01

**Authors:** Song Chen, Changhua Liu, Yuanmin Hao, Yueyue Liu, Xu Liu, Chao Du

**Affiliations:** 1 Key Laboratory of Integrated Crop Pest Management in Southwest China (Ministry of Agriculture), Institute of Plant Protection, Sichuan Academy of Agricultural Sciences, Chengdu, China Institute of Plant Protection, Sichuan Academy of Agricultural Sciences Chengdu China; 2 Institute of Quality Standard and Testing Technology Research, Sichuan Academy of Agricultural Sciences, Chengdu, China Institute of Quality Standard and Testing Technology Research, Sichuan Academy of Agricultural Sciences Chengdu China; 3 Baotou Teachers College, Baotou, China Baotou Teachers College Baotou China

**Keywords:** Genome feature, meloid, mitogenome, oil beetle, phylogenetic relationship

## Abstract

Oil beetles are meloids, which are characterised for their cleptoparasitic habits in bee nests and oily fluid of cantharidin that causes blistering and swelling of the skin. The complete mitochondrial genome of *Meloeproscarabaeus* is determined using the next-generation sequencing technology and its genomic characteristics are described. The 15,653-bp long genome is a circular molecule consisting of 13 protein-coding genes (PCG), 22 transport RNA, two ribosomal RNA, and a control region. The A + T bias of the mitochondrial genome is manifested in the complete sequence and the codon usage of protein-coding genes. The genetic distance within and between genera is calculated to confirm the taxonomic status of *M.proscarabaeus*. The phylogenetic relationships among 15 available meloid taxa are inferred by the maximum likelihood (ML) method based on 13 mitochondrial PCGs. The ML trees resulting from nucleotide and amino acid datasets recover both the monophyly of *Meloe* and *Epicauta* and the polyphyly comprising *Hycleus* and *Mylabris*. This study provides the first description of a mitochondrial genome belonging to the genus *Meloe*. The mitochondrial genome sequence and its characteristics are expected to be conducive to future studies on taxonomy, systematics, and molecular phylogenetics of the family Meloidae.

## ﻿Introduction

The oil beetle *Meloeproscarabaeus* Linnaeus, 1758 is a characteristic species of the meloid genus *Meloe* Linnaeus, 1758, which comprises about 155 species in 16 subgenera and is mainly distributed in the Holarctic region (Sánchez-Vialas et al. 2021; Pan and Bologna 2021). Oil beetles are well known for the oily fluid of hemolymph released from their leg joints, which contains the poison cantharidin that causes blistering and swelling of the skin (Muzzi et al. 2020; Du et al. 2021). Additionally, oil beetles are distinguished by their hypermetamorphic development and cleptoparasitic habits in bee nests (Saul-Gershenz and Millar 2006; Saul-Gershenz et al. 2018).

The taxonomy and phylogeny of the genus *Meloe* are based on morphological characteristics and molecular data (Di Giulio et al. 2002, 2014; Muzzi et al. 2020; Pan and Bologna 2021; Sánchez-Vialas et al. 2021). The genus is considered monophyletic, but more detailed molecular phylogenetic studies are necessary to solve the phylogenetic relationships within the genus. With the simple genetic structure, the high rate of evolution, and the advantage in acquiring methods, the mitochondrial genome has been used widely in many phylogenetic studies of animals (Avise 1994; Cameron 2014; Du et al. 2020). There are 15 meloid species in five genera that have had their complete mitochondrial genomes published in the GenBank database. Previous studies have utilised mitochondrial genome sequences to infer the phylogeny of Meloidae, but without data on the genus *Meloe* due to the absence of mitochondrial genomes for the genus (Du et al. 2017; Liu et al. 2020).

In this study, we determine the complete mitochondrial genome of *M.proscarabaeus* using next-generation sequencing, and we describe its genomic characteristics. Furthermore, we reconstruct the phylogenetic trees based on the nucleotide and amino acid sequences from all available mitochondrial genomes to analyse the phylogenetic relationships among the family Meloidae. The sequence and phylogenetic inferences of *M.proscarabaeus* mitochondrial genome will be a signiﬁcant increase in furthering the study on coleopteran mitochondrial genome architecture and phylogenetics.

## ﻿Materials and methods

### ﻿Sample and genomic DNA extraction

The adults of *Meloeproscarabaeus* were collected from a hill slope in Dongsheng, Inner Mongolia, China (39°45.33'N, 110°01.83'E). The fresh samples were immediately preserved in 100% ethanol and stored in a −20 °C refrigerator. We identified the specimens according to the morphological characters that descried by Pan and Bologna (2021). Total genomic DNA was extracted from a frozen adult using Tianamp Genomic DNA kit following the manufacturer’s protocol. The quality of DNA was determined using 1% agarose gel electrophoresis.

### ﻿Next-generation sequencing and genomic assembly

The library was constructed using an Illumina TruSeq Library Preparation kit with an insert size of 250 bp and sequenced using the paired-end strategy on an Illumina HiSeq 2500 platform. A total of 4.4 Gb raw data was yielded with an average read length of 150 bp. The raw data were trimmed and filtered using fastp with parameters of phred quality ≥ 30 and unqualified percent < 20 to remove adapters and low-quality reads (Chen et al. 2018). Then the clean data were used to assemble the mitochondrial genome of *M.proscarabaeus* by MITObim v. 1.9.1 with the default settings (Hahn et al. 2013). The mitochondrial genome of *Lyttacaraganae* (GenBank accession number NC_033339.1; Du et al. 2017) was employed as a reference sequence. The assembled sequence was aligned with other meloid mitochondrial genomes described by Du et al. (2017) to ensure the assembling quality.

### ﻿Gene annotation and sequence analysis

The complete mitochondrial genome of *M.proscarabaeus* was automatically annotated by the software MitoZ (Meng et al. 2019) and manually compared with other meloid mitochondrial genomes. Of these, PCGs were checked by the identification of open reading frames and aligning with mitochondrial PCGs of other meloids. The annotated mitochondrial genome was analysed its genome characteristics, including nucleotide composition, the composition of skewness, codon usages, and relative synonymous codon usage (RSCU), using MEGA6 (Tamura et al. 2013). All available mitochondrial genomes of 15 meloid species were used to calculate the genetic distances within and between genera in Meloidae. *P*-distances were calculated using MEGA6 with the bootstrap method of 1,000 replications for the variance estimation.

### ﻿Phylogenetic analysis

To infer the phylogenetic relationships among the family Meloidae, all available mitochondrial genomes of 15 meloid species, including the *M.proscarabaeus*, were employed to reconstruct the maximum-likelihood (ML) trees with the *Triboliumcastaneum* (GenBank accession number NC_003081.2; Friedrich and Muqim 2003) as the outgroup. According to GenBank annotations, the nucleotide and amino acid sequences of 13 mitochondrial PCGs from each species were extracted and stop codons removed. Orderly combined sequences were aligned using MAFFT with the default settings (Katoh and Standley 2013) and the gaps and ambiguous sites removed to concatenate into consensus sequences including a 10,934 bp nucleotide and a 3,633 amino acid dataset, respectively. IQ-Tree was employed to estimate the best-fit models of partitioning schemes (Nguyen et al. 2015). The nucleotide and the corresponding amino acid datasets were partitioned for 13 genes individually. The best-fitting models of partitioning schemes were selected with the greedy search algorithm, under the Bayesian information criterion. The ML trees of both datasets were also reconstructed using IQ-Tree with 1000 bootstraps to assess the node support.

## ﻿Results

### ﻿Genome structure and composition

The 5.4 Gb raw data was yielded by the next-generation sequencing with 36,048,728 reads, and the 4.9 Gb (95.06%) clean data was obtained after filtering. The complete mitochondrial genome of *M.proscarabaeus* was assembled using 1,160,002 reads, and the average depth of coverage was assessed at 7,635.95 X. The mitochondrial genome was annotated and then submitted to the GenBank under the accession number OL840851. The complete mitochondrial genome of *M.proscarabaeus* is 15,653 bp in length, which is of moderate length among mitochondrial genomes within Meloidae (Du et al. 2016, 2017; Zhou et al. 2021).

**Figure 1.**
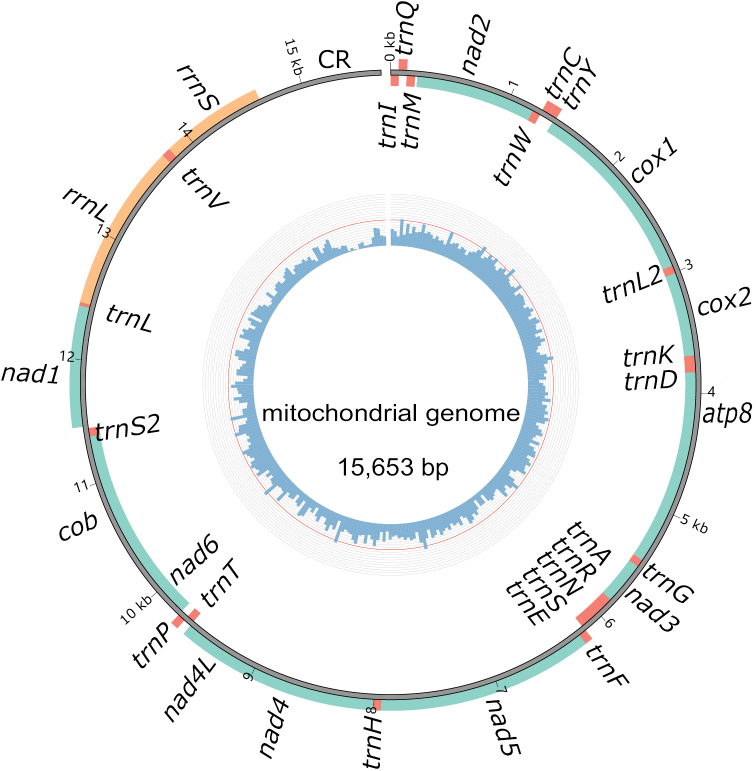
The circular structure of the mitochondrial genome of *Meloeproscarabaeus*. The inner circle represents GC content (the ratio of guanine to cytosine), the outer circle represents genetic characteristics, orange represents ribosomal RNA, red represents transport RNA, and green represents protein-coding regions. Genes in the inner circle (on the J chain) are transcribed clockwise, while those outside the circle (on the N chain) are transcribed counter-clockwise.

The complete mitochondrial genome of *M.proscarabaeus* is a circular DNA molecule consisting of 13 protein-coding genes, 22 transport RNAs, two ribosomal RNAs, and a control region (Fig. 1). The length of *rrnL* and *rrnS* were determined to be 1,329 bp and 816 bp, respectively, and the control region was 1,013 bp in length. The nucleotide base composition of the mitochondrial genome was 37.5% A, 31.6% T, 19.1% C, and 11.9% G. The total A + T content was 69.1%, and the AT skew was 0.0854.

### ﻿Protein-coding gene and codon usage

The total length of the 13 mitochondrial protein-coding genes in *M.proscarabaeus* is 11,127 bp, accounting for 71.10% of the total length of the genome, encoding 3,711 codons in total. All 13 protein-coding genes start using regular initiation codons, including ATT and ATG, and ATA (Table 1), which were commonly used as start codons in insect mitochondrial genomes. Most protein-coding genes terminated with conventional stop codons (such as TAA or TAG), except *cox3*, *nad5*, and *nad4* stopped with T (Table 1).

**Table 1.** Annotation of the *Meloeproscarabaeus* mitogenome.

Gene	Location	Inc	Size	Strand	Anticodon	Start codon	Stop codon
*trnI*	1–66		66	J	GAU		
*trnQ*	64–132	-3	69	N	UUG		
*trnM*	132–201	-1	70	J	CAU		
*nad2*	220–1215	18	996	J		ATT	TAA
*trnW*	1214–1279	-2	66	J	UCA		
*trnC*	1279–1341	-1	63	N	GCA		
*trnY*	1344–1408	2	65	N	GUA		
*cox1*	1401–2948	-8	1548	J		ATT	TAA
*trnL2*	2944–3007	-5	64	J	UAA		
*cox2*	3008–3695	0	688	J		ATA	T*
*trnK*	3696–3766	0	71	J	CUU		
*trnD*	3767–3831	0	65	J	GUC		
*atp8*	3832–3993	0	171	J		ATT	TAA
*atp6*	3984–4655	-10	672	J		ATG	TAA
*cox3*	4655–5437	-1	783	J		ATG	TAA
*trnG*	5441–5503	3	63	J	UCC		
*nad3*	5504–5857	0	354	J		ATA	TAG
*trnA*	5856–5918	-2	64	J	UGC		
*trnR*	5919–5982	0	64	J	UCG		
*trnN*	5983–6049	0	67	J	GUU		
*trnS*	6050–6108	0	59	J	UCU		
*trnE*	6109–6169	0	61	J	UUC		
*trnF*	6168–6231	-2	64	N	GAA		
*nad5*	6232–7942	0	1711	N		ATT	T*
*trnH*	7943–8006	0	64	N	GUG		
*nad4*	8007–9339	0	1333	N		ATG	T*
*nad4l*	9333–9620	-7	288	N		ATG	TAA
*trnT*	9623–9685	2	63	J	UGU		
*trnP*	9686–9748	0	63	N	UGG		
*nad6*	9751–10242	2	492	J		ATT	TAA
*cob*	10242–11381	-1	1140	J		ATG	TAA
*trnS2*	11380–11447	-2	68	J	UGA		
*nad1*	11465–12415	17	951	N		ATT	TAG
*trnL*	12416–12479	0	65	N	UAG		
*rrnL*	12442–13769	-38	1328	N			
*trnV*	13757–13825	-13	69	N	UAC		
*rrnS*	13824–14638	-2	815	N			
CR	14639–15653	0	1015	J			

Inc: intergenic nucleotides, negative values refer to overlapping nucleotides. *trnL2* and *trnS2* refer to *trnL* (UAA) and *trnS* (UGA), respectively. *TAA stop codon is completed by the addition of 3’ A residues to the mRNA.

**Figure 2.**
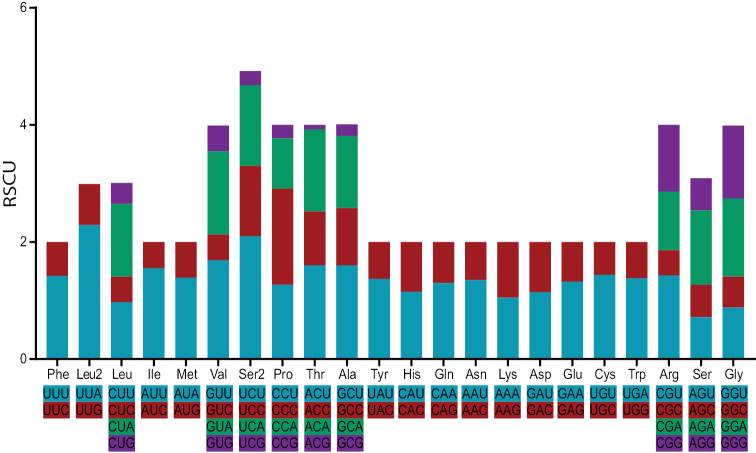
Relative synonymous codon usage (RSCU) in the *Meloeproscarabaeus* mitochondrial genome. An average of 3,711 codons were analysed, excluding stop codons. Codon families are provided on the x-axis. Leu, Leu2, Ser, and Ser2 indicate *trnL1* (CUN), *trnL2* (UUR), *trnS1* (AGN), and *trnS2* (UCN), respectively.

The A + T bias is also manifested in the codon usage of protein-coding genes (Fig. 2). Relatively synonymous codon usages, excluding stop codons, showed that the third position of synonymous codons always has more frequency with A or T than G or C. Additionally, the first three frequently used codons UUA (Leu2), UCU (Ser2), GUU (Val), and some other frequently used codons, including AUU (Ile), UUU (Phe) AAU (Asn), etc. are comprised of two or three A and/or T nucleotides.

### ﻿RNAs and control regions

All 22 transfer RNAs were annotated in the mitochondrial genome of *M.proscarabaeus*. Their length ranged from the shortest *trnS* with 59 bp to the longest *trnK* with 71 bp (Table 1). The maldistribution of transfer RNAs was also found in the mitochondrial genome. For example, there are two clusters comprising six transfer RNAs (*trnA-trnR-trnN-trnS-trnE-trnF*) between *nad3* and *nad5* genes and three transfer RNAs (*trnW-trnC-trnY*) between *nad2* and *cox1*. The remaining RNAs are dispersed among other genes in a single or double way (Fig. 1). Two ribosomal RNAs mitochondrial genome of *M.proscarabaeus* were aligned with these of other meloid and assigned to the blanks between neighbouring genes. The *rrnL* gene was located between *trnL* and *trnV* with a length of 1,328 bp, the *rrnS* was located between *tnrV* and the control region with a length of 815 bp (Table 1). The control region was located between *rrnS* and *trnI* with 1,015 bp in length (Table 1; Fig. 1).

### ﻿Genetic distances

**Figure 3.**
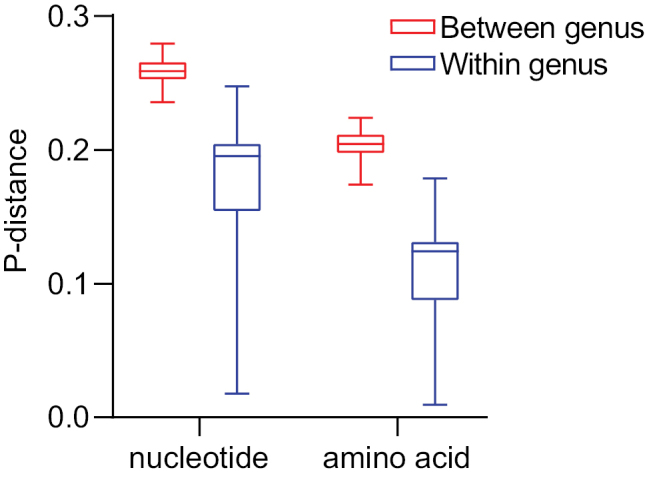
Genetic distance within and between genera. Each boxplot represents the *p*-distance based on the nucleotide and the amino acid datasets from 13 mitochondrial PCGs. The lower horizontal bar represents the smallest observation, the lower edge of the rectangle represents the 25 percentile, the central bar within the rectangle represents the median, the upper edge of the rectangle represents 75 percentile, and the upper horizontal bar represents the largest observation.

The genetic distances within and between genera were calculated using the nucleotide and amino acid data of 13 mitochondrial PCGs among 15 meloid taxa. The result from the nucleotide data showed that the *p*-distances within genera for *Hycleus*, *Epicauta*, and *Mylabris* are 0.167, 0.173, and 0.232, respectively, with an average of 0.191, while the *p*-distances between genera ranged from 0.234 to 0.281 with an average of 0.258 (Fig. 3). The result from the amino acid data showed that the *p*-distances within genus of these three genera are 0.124, 0.107, and 0.163, respectively, with the mean of 0.115, and the *p*-distance between genera ranging from 0.173 to 0.226 with the mean of 0.187 (Fig. 3). The *p*-distances within genus are significantly lower than between genera from both datasets (*p* < 0.01). The *p*-distance between *M.proscarabaeus* and *M.poggii* was 0.116 and 0.064 from the nucleotide and the amino acid data, and far less than the corresponding distances between genera.

### ﻿Phylogenic relationship

The phylogenetic relationships within the family Meloidae were inferred by using maximum likelihood methods, based on the nucleotide and amino acid data from mitogenomes of 15 meloid taxa. The best-fit partitioning schemes and corresponding substitution models are shown in Table 2. Log likelihoods of consensus trees constructed from 1000 bootstrap trees are −88,585.1106 and −32,785.8864 for the nucleotide and amino acid data, respectively.

**Table 2.** The best-fit schemes and evolutionary models for two datasets from mitochondrial genomes.

Dataset	Subset	Best model	Partition names
nucleotide	1	TIM+F+I+G4	*nad2*
	2	GTR+F+I+G4	*cox1*
	3	HKY+F+I+G4	*cox2*, *apt6*, *nad3*, *nad4*, *nad4l*, *nad6*
	4	TPM3+F+G4	*apt8*
	5	TIM2+F+I+G4	*cox3*, *cob*
	6	TPM3+F+I+G4	*nad5*
	7	K3Pu+F+I+G4	*nad1*
Amino acid	1	mtMet+G4	*nad2*, *cox2*, *apt8*, *nad3*, *nad6*
	2	mtZOA+G4	*cox1*
	3	mtVer+G4	*apt6*
	4	mtMAM+G4	*cox3*
	5	mtInv+I+G4	*nad5*
	6	mtInv+G4	*nad4*, *nad4l*
	7	mtMAM+I+G4	*cob*
	8	mtART+G4	*nad1*

**Figure 4.**
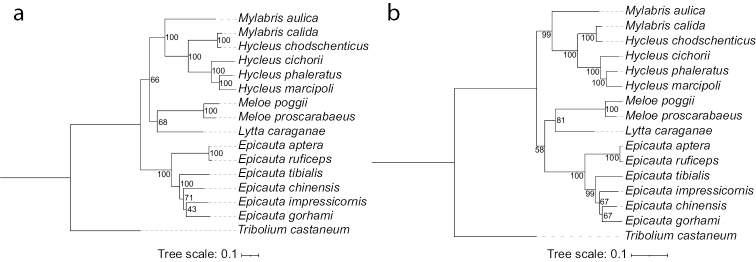
Phylogenetic trees of 14 meloid species based on the nucleotide (a) and amino acid (b) dataset inferred from the maximum likelihood. The numbers abutting branches refer to the bootstrap supports. *Triboliumcastaneum* (Tenebrionidae) was used to root the trees as an outgroup.

The ML trees resulting from both datasets strongly support the monophyly of *Meloe* and *Epicauta*, whereas the genera *Hycleus* and *Mylabris* are not monophyletic (Fig. 4). The monophyletic *Meloe*, including *M.proscarabaeus* and *M.poggii*, sisters with *Lytta* into a branch in both phylogenetic trees, but the branch clusters with *Epicauta* or *Hycleus* in the ML tree from nucleotide or amino acid data, respectively (Fig. 4).

## ﻿Discussion

The first mitochondrial genome of an oil beetle *Meloeproscarabaeus* was sequenced and annotated in this study. The gene arrangement and orientation were the same as the common ancestor for the Insecta (Boore et al. 1998; Taanman 1999). The A + T content and AT skew of *M.proscarabaeus* is the moderate level within Meloidae, and the biased usage of A and T nucleotides is also exhibited in mitochondrial PCGs, because the A + T bias is a common phenomenon in insect mitochondrial genomes (Du et al. 2017). Incomplete stop codons commonly exist in mitochondrial genomes of insects (Du et al. 2016; 2017). It is usual that the single T was employed as the stop codon in many insect mitochondrial genomes, and the incomplete stop codon could be functional in polycistronic transcription cleavage and polyadenylation processes (Ojala et al. 1981). The control region was located between *rrnS* and *trnI* with 1,015 bp in length, which is similar to that of other meloid mitochondrial genomes (Du et al. 2017). It is a noncoding region and a functional region that controls the replication and transcription of the mitochondrial genome and is the biggest region in which variations occurred to affect both sequence and the length of the entire mitochondrial genome (Andrews et al. 1999).

The genetic distance within and between genera was calculated to confirm the taxonomic status of *M.proscarabaeus*. The *p*-distances within genus are significantly lower than between genera from both datasets (*p* < 0.01), but the *p*-distance within *Mylabris* was a little higher than that between *Hycleus* and *Mylabris* (Fig. 3), which may be because of too a few *Mylabris* taxa and also discussed in some fishes and birds (Ma et al. 2020; Du et al. 2020). The *p*-distances resulting from the nucleotide and the amino acid data between *M.proscarabaeus* and *M.poggii* were both far less than the corresponding distances between genera. This indicates there exist a significant distinction in genetic distance within and between genera, and subsequently confirm the taxonomic status of *M.proscarabaeus*.

Phylogenetic analysis within Meloidae recover the monophyly of *Meloe* and *Epicauta*, and a polyphyly comprising *Hycleus* and *Mylabris*. Within the polyphyly, both trees based on the nucleotide and the amino acid datasets show that *M.calida* Pallas, 1782 clustered with *H.chodschenticus* Ballion, 1878 rather than *M.aulica* Menetries, 1832 (Fig. 4). The polyphyly of Mylabrini was also recovered by other studies utilising partial genes (mitochondrial 16S and nuclear ITS2 sequences) and complete mitochondrial PCGs (Bologna et al. 2008; Du et al. 2017). It may be caused by the high similarity between *Hycleus* and *Mylabris* and the inadequate number of taxa available for molecular phylogenetic studies. Previous studies also recovered the different topology (Bologna et al. 2008; Du et al. 2017), which might be limited by the lack of enough taxon sampling. To date, no phylogeny has definitively inferred the phylogenetic relationships within the families. In consideration of the diverse meloid species, the increasingly published information would help achieve more convincing conclusions for the phylogeny of the family.

## ﻿Conclusion

The mitochondrial genome of *M.proscarabaeus* was assembled and described. The genome descriptions provide an informative reference for mitochondrial genomes of *Meloe* beetles. The mitochondrial genome sequence and its characteristics would be beneficial for future studies on taxonomy, molecular phylogenetics, and systematics of meloid insects.

## References

[B1] Andrews RM Kubacka I Chinnery PF Lightowlers RN Turnbull DM Howell N (1999) Reanalysis and revision of the Cambridge reference sequence for human mitochondrial DNA. Nature Genetics 23(2): 147–147. 10.1038/13779 10508508

[B2] Avise JC (1994) Molecular Markers, Natural History and Evolution. Springer Science & Business Media, New York/Philadelphia, 511 pp. 10.1007/978-1-4615-2381-9

[B3] Bologna MA Oliverio M Pitzalis M Mariottini P (2008) Phylogeny and evolutionary history of the blister beetles (Coleoptera, Meloidae). Molecular Phylogenetics and Evolution 48(2): 679–693. 10.1016/j.ympev.2008.04.019 18514547

[B4] Boore JL Lavrov DV Brown WM (1998) Gene translocation links insects and crustaceans. Nature 392(6677): 667–668. 10.1038/33577 9565028

[B5] Cameron S (2014) How to sequence and annotate insect mitochondrial genomes for systematic and comparative genomics research. Systematic Entomology 39(3): 400–411. 10.1111/syen.12071

[B6] Chen S Zhou Y Chen Y Gu J (2018) fastp: An ultra-fast all-in-one FASTQ preprocessor. Bioinformatics (Oxford, England) 34(17): i884–i890. 10.1093/bioinformatics/bty560 PMC6129281 30423086

[B7] Di Giulio A Bologna MA Pinto JD (2002) Larval morphology of the MeloesubgenusMesomeloe: Inferences on its phylogenetic position and a first instar larval key to the *Meloe* subgenera (Coleoptera, Meloidae). The Italian Journal of Zoology 69(4): 339–344. 10.1080/11250000209356479

[B8] Di Giulio A Carosi M Khodaparast R Bologna MA (2014) Morphology of a new blister beetle (Coleoptera, Meloidae) larval type challenges the evolutionary trends of phoresy-related characters in the genus *Meloe*. Entomologia 2: 164. 10.4081/entomologia.2014.164

[B9] Du C He S Song X Liao Q Zhang X Yue B (2016) The complete mitochondrial genome of *Epicautachinensis* (Coleoptera: Meloidae) and phylogenetic analysis among coleopteran insects. Gene 578(2): 274–280. 10.1016/j.gene.2015.12.036 26707213

[B10] Du C Zhang L Lu T Ma J Zeng C Yue B Zhang X (2017) Mitochondrial genomes of blister beetles (Coleoptera, Meloidae) and two large intergenic spacers in *Hycleus* genera. BMC Genomics 18(1): e698. 10.1186/s12864-017-4102-y PMC5585954 28874137

[B11] Du C Liu L Liu Y Fu Z (2020) The complete mitochondrial genome of the Eurasian wryneck *Jynxtorquilla* (Aves: Piciformes: Picidae) and its phylogenetic inference. Zootaxa 4810(2): 351–360. 10.11646/zootaxa.4810.2.8 33055901

[B12] Du C Li W Fu Z Yi C Liu X Yue B (2021) De novo transcriptome assemblies of *Epicautatibialis* provide insights into the sexual dimorphism in the production of cantharidin. Archives of Insect Biochemistry and Physiology 106(4): e21784. 10.1002/arch.21784 33719055

[B13] Friedrich M Muqim N (2003) Sequence and phylogenetic analysis of the complete mitochondrial genome of the flour beetle *Triboliumcastanaeum*. Molecular Phylogenetics and Evolution 26(3): 502–512. 10.1016/S1055-7903(02)00335-4 12644407

[B14] Hahn C Bachmann L Chevreux B (2013) Reconstructing mitochondrial genomes directly from genomic next-generation sequencing reads – A baiting and iterative mapping approach. Nucleic Acids Research 41(13): e129. 10.1093/nar/gkt371 PMC3711436 23661685

[B15] Katoh K Standley DM (2013) MAFFT multiple sequence alignment software version 7: Improvements in performance and usability. Molecular Biology and Evolution 30(4): 772–780. 10.1093/molbev/mst010 23329690 PMC3603318

[B16] Liu YY Zhou ZC Chen XS (2020) Characterization of the complete mitochondrial genome of *Epicautaimpressicornis* (Coleoptera: Meloidae) and its phylogenetic implications for the Infraorder Cucujiformia. Journal of Insect Science 20(2): 16. 10.1093/jisesa/ieaa021 PMC7164779 32302386

[B17] Ma Q He K Wang X Jiang J Zhang X Song Z (2020) Better resolution for Cytochrome b than Cytochrome c Oxidase subunit I to identify *Schizothorax* species (Teleostei: Cyprinidae) from the Tibetan Plateau and its adjacent area. DNA and Cell Biology 39(4): 579–598. 10.1089/dna.2019.5031 32069124

[B18] Meng G Li Y Yang C Liu S (2019) MitoZ: A toolkit for animal mitochondrial genome assembly, annotation and visualization. Nucleic Acids Research 47(11): e63–e63. 10.1093/nar/gkz173 PMC6582343 30864657

[B19] Muzzi M Di Giulio A Mancini E Fratini E Cervelli M Gasperi T Mariottini P Persichini T Bologna MA (2020) The male reproductive accessory glands of the blister beetle *Meloeproscarabaeus* Linnaeus, 1758 (Coleoptera: Meloidae): Anatomy and ultrastructure of the cantharidin-storing organs. Arthropod Structure & Development 59: e100980. 10.1016/j.asd.2020.100980 32829176

[B20] Nguyen LT Schmidt HA Von Haeseler A Minh BQ (2015) IQ-TREE: A fast and effective stochastic algorithm for estimating maximum-likelihood phylogenies. Molecular Biology and Evolution 32(1): 268–274. 10.1093/molbev/msu300 25371430 PMC4271533

[B21] Ojala D Montoya J Attardi G (1981) tRNA punctuation model of RNA procession in human mitochondria. Nature 290(5806): 470–474. 10.1038/290470a0 7219536

[B22] Pan Z Bologna MA (2021) Morphological revision of the Palaearctic species of the nominate subgenus Meloe Linnaeus, 1758 (Coleoptera, Meloidae), with description of ten new species. Zootaxa 5007(1): 1–74. 10.11646/zootaxa.5007.1.1 34810573

[B23] Sánchez-Vialas A Recuero E Jiménez-Ruiz Y Ruiz JL Marí-Mena N García-París M (2021) Phylogeny of Meloini blister beetles (Coleoptera, Meloidae) and patterns of island colonization in the Western Palaearctic. Zoologica Scripta 50(3): 358–375. 10.1111/zsc.12474

[B24] Saul-Gershenz LS Millar JG (2006) Phoretic nest parasites use sexual deception to obtain transport to their host’s nest. Proceedings of the National Academy of Sciences of the United States of America 103(38): 14039–14044. 10.1073/pnas.0603901103 16966608 PMC1599908

[B25] Saul-Gershenz L Millar JG McElfresh JS Williams NM (2018) Deceptive signals and behaviors of a cleptoparasitic beetle show local adaptation to different host bee species. Proceedings of the National Academy of Sciences of the United States of America 115(39): 9756–9760. 10.1073/pnas.1718682115 30201716 PMC6166802

[B26] Taanman JW (1999) The mitochondrial genome: Structure, transcription, translation and replication. Biochimica et Biophysica Acta 1410(2): 103–123. 10.1016/S0005-2728(98)00161-3 10076021

[B27] Tamura K Stecher G Peterson D Filipski A Kumar S (2013) MEGA6: Molecular Evolutionary Genetics Analysis version 6.0. Molecular Biology and Evolution 30(12): 2725–2729. 10.1093/molbev/mst197 24132122 PMC3840312

[B28] Zhou Z Liu Y Chen X (2021) Structural features and phylogenetic implications of three new mitochondrial genomes of blister beetles (Coleoptera: Meloidae). Journal of Insect Science 21(6): e19. 10.1093/jisesa/ieab100 PMC8670627 34905604

